# The Effect of DA-9701 on 5-Hydroxytryptamine-Induced Contraction of Feline Esophageal Smooth Muscle Cells

**DOI:** 10.3390/molecules19045135

**Published:** 2014-04-22

**Authors:** Kyung Hoon Oh, Yoonjin Nam, Ji Hoon Jeong, In Kyeom Kim, Uy Dong Sohn

**Affiliations:** 1 Department of Pharmacology, College of Pharmacy, Chung-Ang University, Seoul 156-756, Korea; E-Mails: okhboss@hanmail.net (K.H.O.); yoonnamjin@naver.com (Y.N.); 2 Department of Pharmacology, School of Medicine, Chung-Ang University, Seoul 156-756, Korea; E-Mail: jhjeong3@cau.ac.kr; 3 Department of Pharmacology, School of Medicine, Kyungpook National University, Daegu 700-422, Korea; E-Mail: inkim@knu.ac.kr

**Keywords:** 5-hydroxytryptamine, feline esophageal smooth muscle, DA-9701, Pharbitidis Semen, Corydalis Tuber, MLC_20_, phosphorylation

## Abstract

Serotonin, or 5-hydroxytryptamine (5-HT), is a monoamine neurotransmitter found in blood platelets, the gastrointestinal (GI) tract, and the central nervous system (CNS) of animals and humans. The signaling pathways of 5-hydroxytryptamine (5-HT)-induced contractions in cat esophageal smooth muscle cell (ESMC)s have been identified, but the downstream components of the 5-HT signaling pathway remain unclear. DA-9701 is the standardized extract of the *Pharbitis nil* Choisy seed (Pharbitidis Semen, Convolvulaceae) and the root of *Corydalis yahusuo* W.T. Wang (Corydalis Tuber, Papaveraceae). DA-9701 is known to have strong gastroprokinetic effects and a good safety profile. In this study, we investigated the 5-HT signaling pathway at the G-protein level, and we explored the mechanisms by which DA-9701 induces smooth muscle contraction. Freshly isolated smooth muscle cells were harvested from the feline esophagus, and cells were permeabilized to measure their length. 5-HT produced esophageal smooth muscle contractions in a dose-dependent manner. Furthermore, 5-HT produced a relatively long-acting contraction. 5-HT binds to the 5-HT_2_, 5-HT_3_ and 5-HT_4_ receptors to induce smooth muscle contraction in feline ESMCs. These receptors, which are located in esophageal smooth muscle, are coupled to G_αq_, G_αo_ and G_αs_. These G proteins activate PLC, which leads to Ca^2+^/calmodulin-dependent MLCK activation, resulting in MLC_20_ phosphorylation and cell contraction. Conversely, DA-9701 inhibits 5-HT-induced contraction by inhibiting MLC_20_ phosphorylation.

## 1. Introduction

Serotonin, or 5-hydroxytryptamine (5-HT), is a monoamine neurotransmitter found in blood platelets, the GI tract and the central nervous system (CNS) of animals and humans [1]. It is a well-known neurotransmitter in the pathways associated with mood, and its action is known to contribute to the feeling of happiness [2].

Approximately 90% of the human body’s total 5-HT is located in enterochromaffin cells in the gut, where it is used to regulate intestinal movement [3,4]. 5-HT has proven to be an important mediator of gastrointestinal motility [5,6]. Based on pharmacological criteria, 5-HT receptors are classified into seven main receptor subtypes. Five of them are present in enteric neurons, enterochromaffin cells, and GI smooth muscle, namely 5-HT_1_, 5-HT_2_, 5-HT_3_, 5-HT_4_ and 5-HT_7_ [7,8]. In the gastrointestinal tract, 5-HT is reported to contribute to the regulation of motility and secretion via several 5-HT receptor types [9]. Serotonin receptors are activated by the neurotransmitter serotonin, which acts as their natural ligand [6], and serotonin receptors are the target of a variety of pharmaceutical and illicit drugs, including many antidepressants, antipsychotics, anorectics, antiemetics, gastroprokinetics, antimigraine agents, hallucinogens and entactogens [10].

G proteins function to transduce the effect of a ligand binding to a cell surface receptor into intracellular signals. Isolated ESMCs were permeabilized with saponin to allow G protein antibodies to enter into the cytoplasm. These antisera, raised against synthetic peptides corresponding to the amino acid sequence of the carboxyl terminal of the G protein α subunit, have been used as effective probes for G protein structure and function [11]. For example, antibodies raised against the carboxyl terminal region of the α subunit of G_αi2_ successfully blocked receptor-mediated adenylyl cyclase inhibition [12].

Many studies have shown diverse mechanisms by which intestinal smooth muscle contraction occurs [13,14]. Contraction of the lower esophageal sphincter by acetylcholine (ACh) is mediated by activation of M_3_ muscarinic receptors linked to G_q_ and to phosphatidylinositol-specific phospholipase C (PI-PLC), as well as by production of inositol 1, 4, 5-trisphosphate (IP_3_) and diacylglycerol (DAG). IP3 causes release of Ca^2+^ from stores at a concentration sufficient to cause activation of calmodulin (CAM), and Ca^2+^ CAM causes activation of myosin light chain kinase (MLC kinase).

Acetylcholine-induced contraction of esophageal muscle is mediated by muscarinic M_2_ receptors linked to G_i3_-type G proteins, which activate phosphatidylcholine-specific phospholipase C (PC-PLC) and phospholipase D (PLD) to produce DAG. DAG and arachidonic acid (AA), which are generated by cytosolic phospholipase A_2_ (cPLA_2_), interact to activate protein kinase C (PKC)-ε. The influx of Ca^2+^ may independently activate the same phospholipases and produce the same second messengers, potentiating the activation of PKC-ε, which, in turn, is linked to two separate mitogen-activated protein (MAP) kinase pathways. One MAP kinase pathway is dependent on ERK1/ERK2, and the other is dependent on heat-shock protein (HSP) 27-linked p38 MAP kinase [15].

In many GI studies, guinea pigs, rats, mice, ferrets, dogs and humans have been commonly used as models [16,17,18,19,20]. However, the effect of 5-HT on the regulation of intestinal contractions has not been established in felines. Likewise, the involvement of 5-HT receptors in 5-HT-elicited responses has been less extensively investigated in the feline esophagus. 

DA-9701 is the standardized extract of the seed of *Pharbitis nil* Choisy (Pharbitidis Semen, Convolvulaceae) and the root of *Corydalis yahusuo* W.T. Wang (Corydalis Tuber, Papaveraceae) [21]. We previously found that DA-9701 has strong gastroprokinetic effects and a safety profile superior to conventional prokinetics like cisapride and mosapride [21]. DA-9701 not only accelerates gastric emptying and the GI transit of meals in normal conditions and conditions that result in an abnormal delay, but also enhances gastric accommodation in conscious dogs [21].

In the present study, the signaling pathways initiated by 5-HT in feline esophageal muscle cells were identified. Also, the effect of DA-9701 on 5-HT-induced contraction was confirmed. In order to determine the receptor mediating the contraction, selective 5-HT agonists and antagonists were used. Specific G protein antibodies were used to identify the coupling of specific G proteins to effector enzymes, and selective inhibitors were used to characterize the pathways involved in MLC_20_ (20 kDa regulatory light chain of myosin II) phosphorylation and muscle contraction. Pretreatment with DA-9701 and then treatment with 5-HT and 5-HT agonists elucidated the effect of DA-9701 on 5-HT-induced contraction, and MLC_20_ phosphorylation was used to determine the effect of DA-9701.

## 2. Results and Discussion

### 2.1. Identification of Dispersed Esmcs

**Figure 1:**
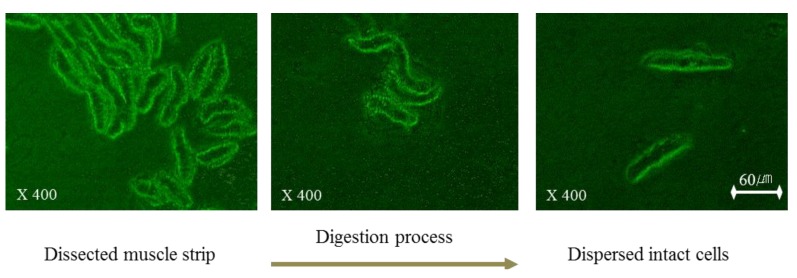
The dispersing process of feline ESMCs.

To assess the dispersing process of esophageal smooth muscle in collagenase buffer, the dissected smooth muscle strip was observed every 5 min by microscopy after an overnight incubation. After this incubation, we observed that ESMCs were separated from the esophageal smooth muscle tissue squares (Figure 1). After identification of the separate ESMCs, the incubation was ended to prepare the dispersed muscle cell suspension. In Figure 1, the dispersing process is shown starting on the left and proceeding to the right. Freshly isolated ESMCs were spindle shaped with diverse lengths, ranging from 38–92 µm. The dispersing process was captured using a digital closed-circuit video camera.

### 2.2. 5-HT Induces Contraction of Isolated ESMCs

**Figure 2:**
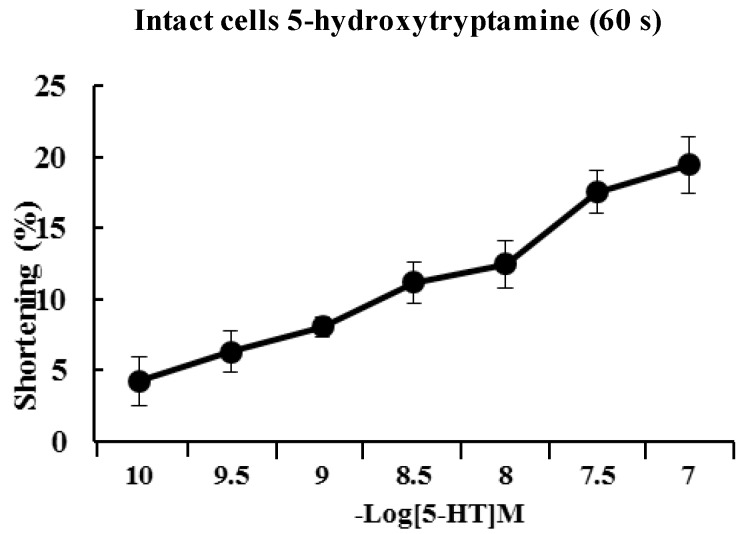
Dose-dependent contractile response of cat ESMCs to 5-HT. Data are expressed as the means ± S.E. of four experiments.

**Figure 3:**
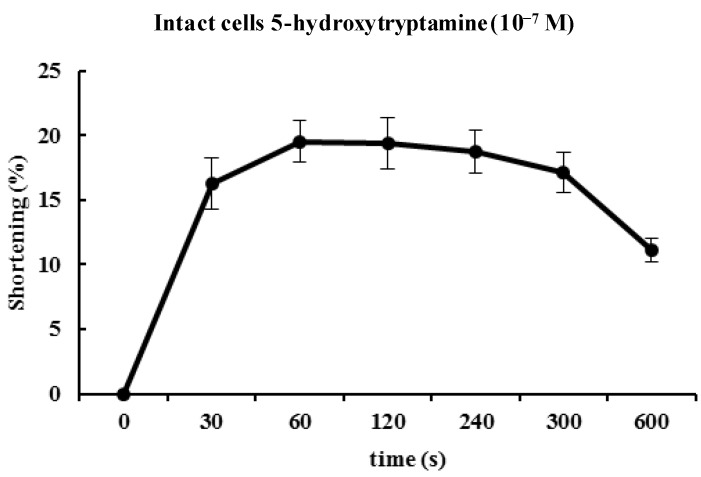
Time course of the contractile response of cat smooth muscle cells to 5-HT (10^−7^ M). Data are expressed as the means ± S.E. of four experiments.

Approximately 90% of the human body’s total serotonin is located within enterochromaffin cells in the gut where it is used to regulate intestinal movements. In the present study, freshly isolated ESMCs were stimulated for 60 s with 10^−1^^0^ to 10^−7^ M 5-HT. The response to 5-HT was concentration-dependent with a maximal response observed at 10^−7^ M (Figure 2). Figure 3 illustrates the time course of 5-HT-induced contractions. The contractions peaked at 60 s and then slowly declined. Based on the concentration-response and time-course data, ESMCs were exposed to 5-HT at a final concentration of 10^−7^ M for 60 s in most of the later experiments (Figure 3).

As mentioned, the contractions showed concentration dependency that reached a maximum at 10^−7 ^ M. The cellular response to 5-HT was evoked quickly (in 60 s) and decreased slowly. 5-HT-induced responses were initiated quickly and had prolonged effects in ESMCs. Next, the mechanism of 5-HT-induced contraction in feline ESMCs was explored.

### 2.3. Effects of a 5-HT Receptor Antagonist on 5-HT-Induced Contraction

To test whether the 5-HT_1_ or 5-HT_2_ receptor is involved in 5-HT-induced muscle contraction, freshly isolated ESMCs were pretreated with (A) methysergide (5-HT_1A_ partial agonist and 5-HT_2B,C_ receptor antagonist at 10^−8^ M, 10^−7^ M, and 10^−6^ M) or (B) ketanserin (a selective 5-HT_2_ receptor antagonist at 10^−8^ M, 10^−7^ M, and 10^−6^ M) for 1 min and then treated with 5-HT (10^−7^ M). Methysergide did not inhibit 5-HT-induced contraction. Pretreatment with ketanserin, on the other hand, significantly inhibited 5-HT-induced smooth muscle cell contraction.

**Figure 4:**
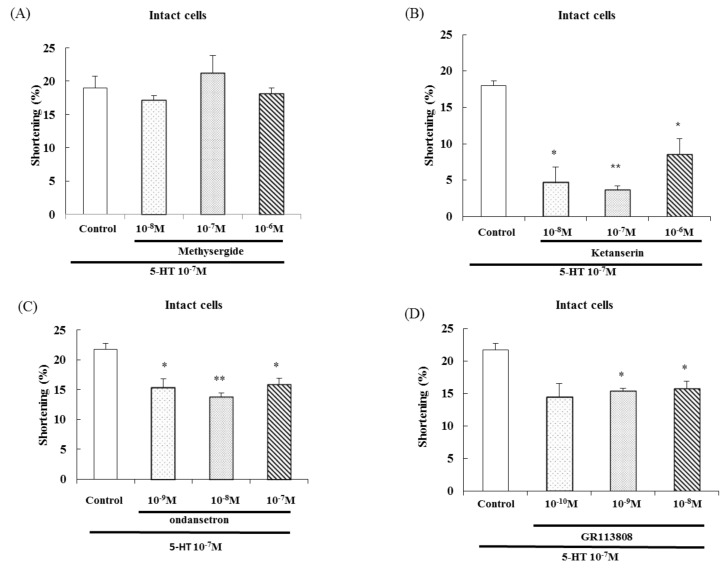
Effect of a 5-HT antagonist on 5-HT-induced contractions in isolated ESMCs. (**A**) Effect of a 5-HT_1_ partial agonist and 5-HT_2_ antagonist on 5-HT-induced contractions; (**B**) Effect of a 5-HT_2_ antagonist on 5-HT-induced contractions; (**C**) Effect of a 5-HT_3_antagonist on 5-HT-induced contractions; (**D**) Effect of a 5-HT_4_ antagonist on 5-HT-induced contractions. Data are expressed as the means ± S.E. of four experiments (Student’s *t*-test; ** *p* < 0.01 *vs.* control, * *p* < 0.05 *vs.* control).

To identify whether the 5-HT_3_ or 5-HT_4_ receptor is involved in 5-HT-induced contraction, freshly isolated ESMCs were pretreated with (C) ondansetron (a selective 5-HT_3_ receptor antagonist at 10^−9^, 10^−8^, and 10^−7^ M) or (D) GR113808 (a selective 5-HT_4_ receptor antagonist at 10^−1^^0^, 10^−9^ and 10^−8^ M) for 1 min and then treated with 5-HT (10^−7^ M). Ondansetron and GR113808 significantly inhibited 5-HT-induced smooth muscle cell contraction. These results suggest that 5-HT_2_, 5-HT_3_ and 5-HT_4_ receptors mediate 5-HT-induced contraction (Figure 4). Based on Figure 4A, 5-HT_1_ receptor seems to be involved in 5-HT-induced contraction. It needs further study to evaluate.

### 2.4. Characterization of the G Protein Subtypes Involved in 5-HT-Induced Contraction

The serotonin receptors, also known as 5-hydroxytryptamine receptors, or 5-HT receptors, are a group of G protein-coupled receptors and ligand-gated ion channels [22]. After specific antibodies bind, G protein cannot be activated by signals from receptors, blocking the transduction of the signal to its effectors. We have previously shown that G_αi1_, G_αi2_, G_αi3_, G_β_ (40 kDa), G_αo_ (40 kDa), G_αq_ (42 kDa), and G_αs_ (46 kDa) proteins exist in cat ESMCs [23].

**Figure 5:**
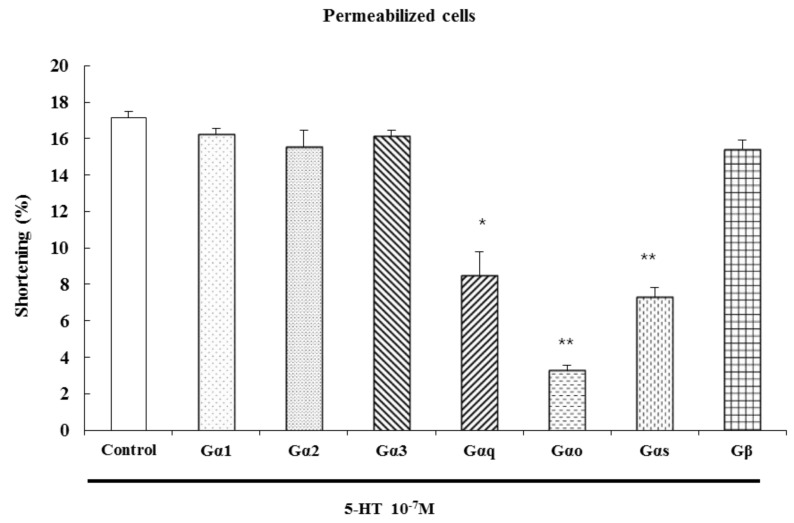
Inhibition of 5-HT-induced contraction in permeabilized ESMCs by antibodies to G protein isoforms. Data are expressed as the means ± S.E. of four experiments (Student’s *t*-test; ** *p* < 0.01 *vs.* control, * *p* < 0.05 *vs.* control).

To identify the specific G protein involved in 5-HT-induced esophageal contraction, cells were permeabilized with saponin in cytosolic medium containing each G protein antibody (1:200 dilution) to allow diffusion of antibodies into the cell. These antibodies block receptor-induced activation of G proteins by binding to their terminal peptide regions, which normally interact with the receptor. Antibodies to G_αq_, G_αo_ and G_αs_ inhibited 5-HT-induced contractions, but antibodies to G_αi1_, G_αi2_, G_αi3_, and G_β_ did not. We firstly confirmed that the antibodies used in these experiments did not affect the contraction of ESMCs by themselves. These data suggest that 5-HT-induced muscle contraction is mediated by G_αq_, G_αo_, and G_αs_ proteins (Figure 5).

### 2.5. Pathways Mediating 5-HT-Induced Smooth Muscle Contraction

In order to investigate whether 5-HT-induced contractions in dispersed ESMCs are associated with PLC and MLCK, ESMCs were pretreated with a PLC inhibitor (U73122, 1 μM) for 10 min and an MLCK inhibitor (ML-9, 10 μM) for 10 min before a 60 s 5-HT treatment. The smooth muscle cell contractions induced by 5-HT were reduced by both the PLC inhibitor, U-73122 (51.2% inhibition), and the MLCK inhibitor, ML-9 (47.8% inhibition).

**Figure 6:**
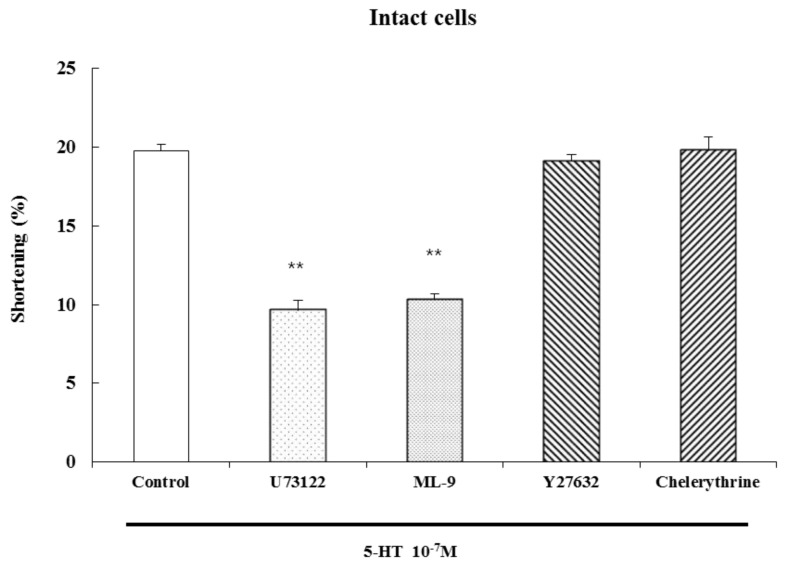
Cat ESMCs contraction induced by 5-HT (10^−7^ M). Data are expressed as the means ± S.E. of four experiments (Student’s *t*-test; ** *p* < 0.01 *vs.* control).

To determine whether 5-HT-induced contraction in dispersed ESMCs is associated with Rho kinase and PKC, ESMCs were pretreated with a Rho kinase inhibitor (Y27632, 1 μM) for 10 min and a PKC inhibitor (chelerythrine, 10 μM) for 1 min before the 60 s 5-HT treatment. The smooth muscle cell contraction induced by 5-HT was not affected by Y27632 or chelerythrine. The inhibitors didn’t affect contractions by themselves. These results suggest that 5-HT-induced contraction is mediated by PLC and MCLK in dispersed ESMCs (Figure 6).

Cell contraction induced by 5-HT was inhibited by the MLC kinase inhibitor, ML-9, but was not affected by the Rho kinase inhibitor, Y27632, or the PKC inhibitor, chelerythrine. These results indicate that smooth muscle cell contraction is Ca^2+^-dependent, requiring IP_3_-dependent Ca^2+^ release and formation of the Ca^2+^-calmodulin complex, which activates MLC kinase.

### 2.6. PLCβ1 Mediates 5-HT-Induced Contraction

**Figure 7:**
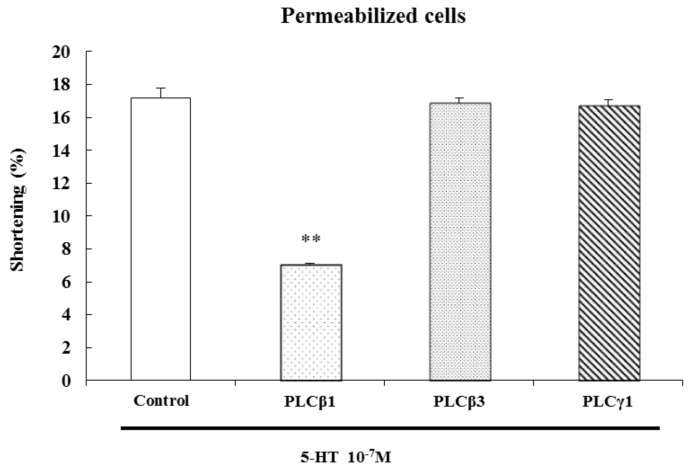
PLC β1 mediated the 5-HT-induced contraction of permeabilized ESMCs*.* Data are expressed as the means ± S.E. of four experiments (Student’s *t*-test; ** *p* < 0.01 *vs.* control).

In the gastrointestinal tract, activated phospholipases produce second messengers like arachidonic acid, inositol-1,4,5-triphosphate (IP_3_), and diacylglycerol (DAG) by degrading phospholipids to induce contraction. In our previous study of dispersed ESMCs, we demonstrated the presence of 150 kDa immunoreactive bands with PLC β1 and PLC β3 antibodies, and we demonstrated a 145-kDa band with a PLC γ1 antibody [23]. To determine whether PLC-mediated contraction is isozyme-specific, the effects of 5-HT-induced contraction were examined. Incubation of permeabilized smooth muscle cells for 1 h with a PLC β1-specific antibody (1:200) inhibited 5-HT-induced contraction. No other PLC-specific antibody had a significant effect on contraction. The antibodies that used in this experiment had no effect on contraction. These results indicate that 5-HT-induced contraction is mediated by PLC β1 (Figure 7). Of the phospholipase inhibitors, the PLC inhibitor, U73122, reduced 5-HT-induced contraction. These data emphasize the role of PLC β1 in mediating esophageal smooth muscle contraction induced by 5-HT.

### 2.7. Effects of DA-9701 on 5-HT-Induced Contraction

**Figure 8:**
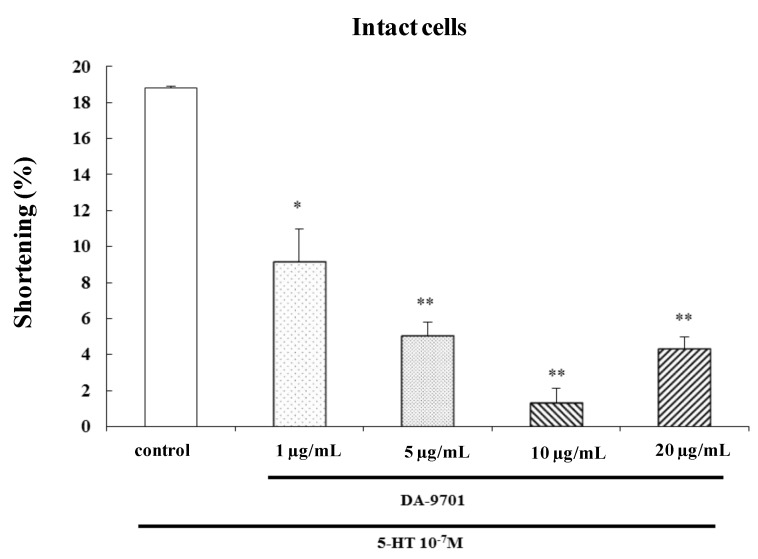
Effect of DA-9701 on 5-HT-induced contraction in isolated ESMCs. Data are expressed as the means ± S.E. of four experiments (Student’s *t*-test; ** *p* < 0.01 *vs.* control, * *p* < 0.05 *vs.* control).

To investigate the effect of DA-9701 on 5-HT-induced contraction, freshly dispersed ESMCs were incubated with DA-9701 (1, 5, 10, and 20 μg/mL) for 1 min and then treated with 5-HT (10^−7^ M). DA-9701 significantly inhibited 5-HT-induced contraction. The most effective dose of DA-9701 was 10 μg/mL (Figure 8).

### 2.8. Effects of DA-9701 on 5-HT Agonist-Induced Contraction

To test the effect of 5-HT receptor agonist-induced contraction, freshly dispersed ESMCs were treated with a 5-HT_2_ agonist (α-methylserotonin, 10^−6^ M), a 5-HT_3_ agonist (2-methyl-5-HT, 10^−6^ M), and a 5-HT_4_ agonist (mosapride, 10^−7^ M). α-Methylserotonin, 2-methyl-5-HT, and mosapride caused feline ESMCs to contract. These results offered additional confirmation that 5-HT_2_, 5-HT_3_, and 5-HT_4_ receptors are involved in 5-HT-induced contraction.

**Figure 9:**
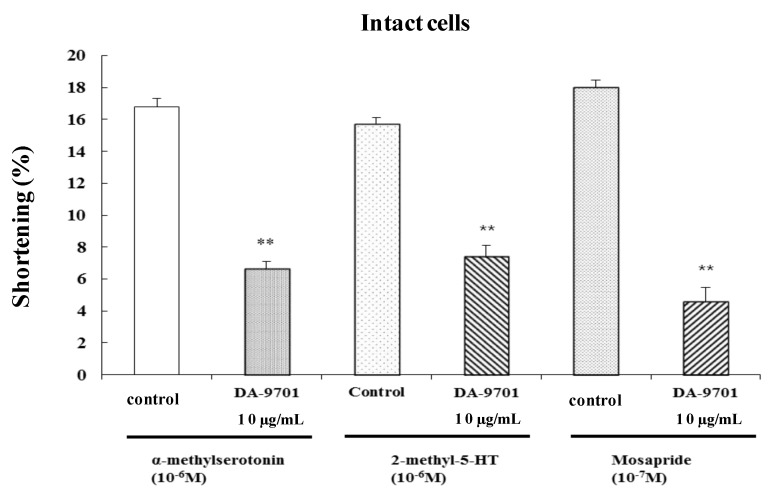
The effect of DA-9701 on 5-HT agonist-induced contraction of cat ESMCs. Data are expressed as the means ± S.E. of four experiments (Student’s *t*-test; ** *p* < 0.01 *vs.* control).

To identify the effect of DA-9701 on 5-HT agonist-induced contraction, freshly dispersed ESMCs were incubated with DA-9701 (10 μg/mL) for 1 min and then treated with a 5-HT_2_ agonist (α-methylseroyonin), a 5-HT_3_ agonist (2-methyl-5-HT), and a 5-HT_4_ agonist (mosapride). Contraction was measured after 1 min. DA-9701 significantly inhibited 5-HT agonist-induced contraction (Figure 9).

### 2.9. Effects of DA-9701 and 5-HT on MLC20 Phosphorylation

**Figure 10:**
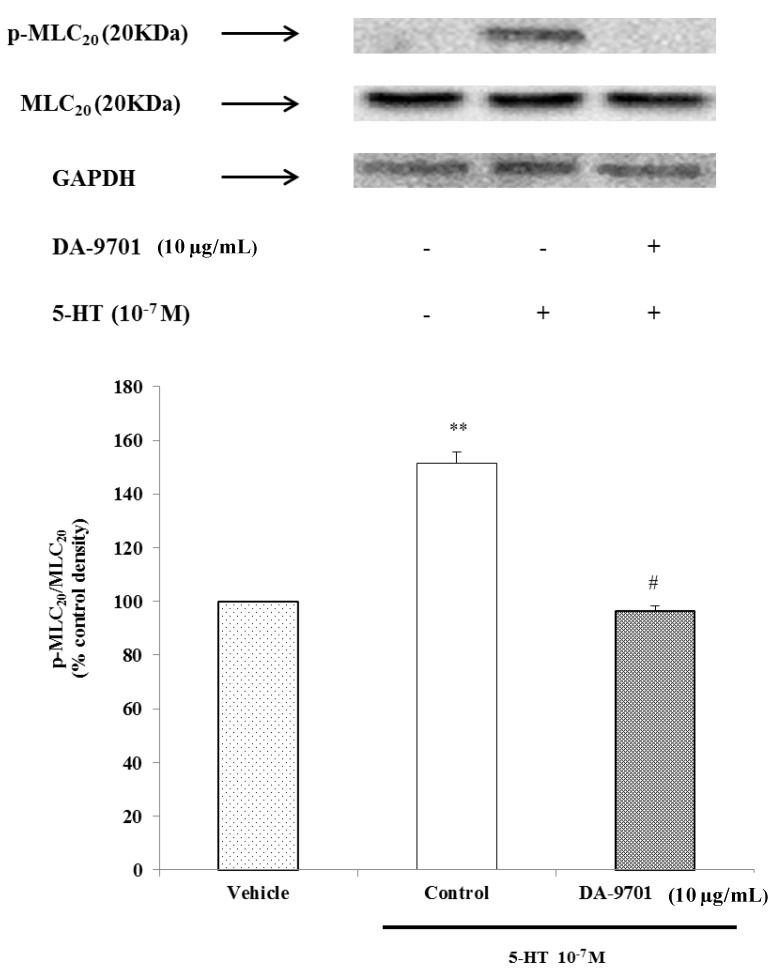
The effect of 5-HT and DA-9701 on phosphorylation of MLC_20_. Data are expressed as the means ± S.E. of four experiments (Student’s *t*-test; ** *p* < 0.01 *vs.* vehicle, ^#^ *p* < 0.01 *vs.* control).

We performed an MLC_20_ phosphorylation experiment to determine whether 5-HT-induced phosphorylation of MLC_20_ occurred at Ser^19^ in dispersed ESMCs, and we observed that 5-HT significantly increased phosphorylation of MLC_20_. To identify the effect of DA-9701 on phosphorylation of MLC_20_ in 5-HT-induced muscle contraction, freshly dispersed ESMCs were pretreated with DA-9701 (10 μg/mL) for 1 min and then treated with 5-HT (10^−7^ M). As shown in Figure 10, the p-MLC_20_/MLC_20_ level decreased in DA-9701 treated cells. These results indicate that DA-9701 significantly inhibits phosphorylation of MLC_20_ (Figure 10). 

It was previously found that DA-9701 has strong gastroprokinetic effects and a safety profile superior to conventional prokinetics, including cisapride and mosapride [21]. ESMCs were exposed to various concentrations of DA-9701 and then treated 5-HT. DA-9701 inhibited 5-HT-induced contraction, and the inhibition was maximal at a treatment dose of 10 μg/mL. Increased immunoreactive protein bands of 5-HT-treated cells corresponded to the 20 kDa phosphospecific Ser_19_-MLC_20_ antibody. These results indicate that activated MLC kinase phosphorylates MLC_20_.

## 3. Experimental

### 3.1. Materials

5-Hydroxytryptamine hydrochloride, methysergide maleate salt, ketanserin (+)-tartrate salt, ondansetron hydrochloride dihydrate, GR113808 (1-[2-[(methylsulfonyl)amino]ethyl]-4-piperidinyl]methyl-1-methyl-1*H*-indole-3-carboxylate), α-methylserotonin, mosapride, U73122, ML-9, Y27632, and chelerythrine were from Sigma Chemical Co. (St Louis, MO, USA). 2-methyl-5-hydroxytryptamine HCl (Tocris, Bristol, UK), DA-9701 (Dong-A Pharmaceutical Co., Seoul, Korea ), G protein antibodies (G_i1_, G_i2_, G_i3_, G_q_, G_s_, G_o_, and G β), PLC isozyme antibodies (β1, β3, γ1), phosphospecific Ser_19_-MLC_20_ antibody, and GAPDH antibody were from Santa Cruz Biotechnology (Santa Cruz, CA, USA) and Ser_19_-MLC_20_ antibody was from Cell Signaling Technology (Beverly, MA, USA). Goat anti-rabbit IgG-HRP and goat anti-mouse IgG-HRP were from Zymed Laboratories Inc. (Eccles Avenue, CA, USA). The rainbow molecular weight marker was from Amersham (Arlington Heights, IL, USA). The enhanced chemiluminescence (ECL) agents were from PerkinElmer Life Sciences (Boston, MA, USA). The sodium dodecyl sulfate (SDS) sample buffer was from Owl Scientific Inc. (Woburn, MA, USA). The nitrocellulose membrane, Tris/Glycine/SDS and Tris/Glycine buffers were from BioRad (Richmond, CA, USA). The phosphate-buffered saline (PBS) was from Roche Diagnostics Co. (Indianapolis, IN, USA). Restore^TM^ Western blot stripping buffer was from Pierce (Rockford, IL, USA). The 4-(2-hydroxyethyl)-1-piperazine-N'-2-ethane sulfonic acid (HEPES), collagenase type F, ammonium persulfate, Ponceau S, bovine serum albumin (BSA), leupeptin, aprotinin, β-mercaptoethanol, *N,N,N',N'*-tetramethylethylene diamine (TEMED), ethylene glycol-bis-(β-aminoethylether)-*N,N,N',N'*-tetraacetic acid (EGTA), ethylene diamine tetraacetic acid (EDTA), phenylmethyl-sulfonylfluoride (PMSF) and other reagents were purchased from Sigma Chemical Co. (St Louis, MO, USA).

### 3.2. Preparation of Dispersed Muscle Cells

Single muscle cells were isolated as previously described [24]. Muscle strips were incubated overnight in normal potassium HEPES buffer containing 1 mg/mL papain, 1 mM dithiothreitol, 1 mg/mL BSA, and 0.5 mg/mL collagenase (type F, Sigma), and muscle strips were equilibrated with 95% O_2_–5% CO_2_ to maintain a pH of 7.0 at 31 °C. The composition of normal potassium HEPES buffer was 1 mM CaCl_2_, 250 µM EDTA, 10 mM glucose, 10 mM HEPES, 4 mM KCl, 131 mM NaCl, 1 mM MgCl_2_, and 10 mM taurine. Twenty-four h later, tissue was warmed to room temperature for 30 min and incubated in a water bath at 31 °C for 30 min. After incubation, the digested tissue was poured over a 360 µm Nitex filter, rinsed in collagenase-free HEPES buffer to remove any trace of collagenase, and then incubated in this solution at 31 °C gassed with 95% O_2_–5% CO_2_. The cells were allowed to dissociate freely for 10 to 20 min. Suspensions of single muscle cells were harvested by filtration through a 500 µm Nitex mesh [24]. Before beginning the experiment, the cells were kept at 31 °C for at least 10 min to relax them. Throughout the procedure, care was taken not to agitate the fluid to avoid cell contraction in response to mechanical stress. Animal experiments were approved by the Institutional Animal Care and Use Committee of Chung-Ang University, in accordance with the guide for the Care and Use of Laboratory Animals in Seoul, Korea.

### 3.3. Preparation of Permeabilized Smooth Muscle Cells

When required, cells were permeabilized to allow the non-diffusible agents, such as G protein antibodies or PLC isozyme antibodies, to diffuse across the intact cell membranes. Notably, contraction in permeabilized cells is not significantly different from that in intact cells [25,26,27,28,29]. After completion of the enzymatic phase of the digestion process, the partly digested muscle tissue was washed with an enzyme-free cytosolic buffer with the following composition: 20 mM NaCl, 100 mM KCl, 5.0 mM MgSO_4_, 0.96 mM NaH_2_PO_4_, 1.0 mM EGTA, 0.48 mM CaCl_2_, and 2% bovine serum albumin. The cytosolic buffer was equilibrated with 95% O_2_–5% CO_2_ to maintain a pH of 7.2 at 31 °C. Muscle cells dispersed spontaneously in this medium. The cytosolic buffer contained 0.48 mM CaCl_2_ and 1 mM EGTA, yielding 0.18 mM of free Ca^2+^ [30]. After dispersion, the cells were permeabilized by incubation for 5 min in cytosolic buffer that contained saponin (75 µg/mL). After exposure to saponin, the cell suspension was centrifuged at 350 g, and the resulting pellet was washed with saponin-free modified cytosolic buffer that contained antimycin A (10 µM), ATP (1.5 mM), and an ATP-regenerating system that consisted of creatine phosphate (5 mM) and creatine phosphokinase (10 units/mL). After the cells were washed free of saponin, they were re-suspended in modified cytosolic buffer.

### 3.4. Preparation of Dispersed Muscle Cells

Contraction of isolated muscle cells was measured by scanning micrometry [31]. An aliquot of cell suspension containing 10^4^ muscle cells/mL was added to HEPES medium containing the test agents. The reaction was terminated by addition of acrolein (1% final concentration). A length of 40 to 50 muscle cells treated with a contractile agent was measured at random by a scanning micrometry phase-contrast microscope (model ULWCD 0.30 Olympus, Tokyo, Japan) and by a digital closed-circuit video camera (CCD color camera, Toshiba, Tokyo, Japan) connected to a Macintosh computer (Apple, Cupertino, CA, USA) using the NIH Image 1.57 software program (National Institutes of Health, Bethesda, MD, USA). Measurements of the treated cells were compared to the lengths of untreated cells. Contraction was expressed as the percent reduction in mean cell length from baseline. Using agonists, the time course of contraction consists of a peak contraction followed by a lower, sustained plateau. In the present study, contraction refers to the one that occurred after 60 s of agonist (5-HT) exposure.

### 3.5. Western Blot Analysis of MLC20 Phosphorylation

Equal amounts of the proteins from each sample were resolved on an SDS-polyacrylamide gel by electrophoresis, and the Bradford reagents determined the supernatant protein concentration, according to the instructions of the manufacturer (Bio-Rad) The absorbance was measured spectrophotometrically at a wavelength of 595 nm.

Phosphorylated MLC_20_ was determined by immunoblot analysis using a phospho-specific antibody [13,32]. Previously frozen dispersed muscle cell samples were homogenized in a buffer containing 20 mM Tris-HCl (pH 7.4), 0.5 mM EDTA, 0.5 mM EGTA, 1% (*w/v*) Triton X-100, 0.01% (*w/v*) SDS, 10 μg/mL leupeptin, 10 μg/mL aprotinin, 1 mM PMSF, and 0.7 μg/mL β-mercaptoethanol. Samples of the homogenates were then centrifuged for 10 min at 4 °C, and the supernatants were collected. Aliquots were subjected to electrophoresis on an SDS-polyacrylamide gel. A pre-stained molecular mass marker was also run in an adjacent lane to allow for molecular mass determination of the loading buffer, which consisted of 25 mM Tris (pH 8.3), 192 mM glycine, and 0.1% SDS. The separated proteins were transferred to a 0.45-μm nitrocellulose membrane in transfer buffer (25 mM Tris (pH 8.3), 192 mM glycine, and 20% (*v/v*) methanol) using a power supply (Power Pac 1000, Bio-Rad, Melville, NY, USA). To confirm gel-loading uniformity, blots were stained with Ponceau S. After confirmation, membranes were washed with PBS and then incubated in PBS buffer containing 5% non-fat dry milk for 1 h at room temperature to block nonspecific binding. After washing with PBS three times for 5 min, the membrane was incubated overnight with antibodies (1:1000 dilution) to MLC_20_ (Ser^19^) in a PBS solution containing 0.1% BSA at 4 °C. Then, the membrane was washed with PBS containing 0.05% Tween 20 twice for 5 min, and then the membrane was incubated with horseradish peroxidase-conjugated secondary antibody (1:5000 dilution) for 1 h at room temperature. Enhanced chemiluminescence agents (ECLs; Perkin Elmer) were used to detect immunoreactive bands, which were developed by an X-ray film developer and fixer. Developed films from ECL were scanned and analyzed densitometrically using Scion Image. Phosphorylation of MLC_20_ was calculated as the ratio of phosphorylated MLC_20_ to total MLC_20_.

### 3.6. Analysis of Data

Data from individual assays represent the mean values from triplicate measurements. The data were expressed as the means ± S.E.M. and analyzed with a Student’s *t*-test. *p* < 0.05 was considered statistically significant. 

## 4. Conclusions

In the present study, 5-HT-induced contraction was mediated by 5-HT_2_, 5-HT_3_, and 5-HT_4_ receptors, as determined by the use of 5-HT antagonists. ESMCs were pretreated with DA-9701, followed by treatment with 5-HT_2_, 5-HT_3_, and 5-HT_4_ agonists. 5-HT_2_, 5-HT_3_, and 5-HT_4_ agonists induced cell contraction, and DA-9701 inhibited those actions. Therefore, 5-HT_2_, 5-HT_3_, and 5-HT_4_ receptors were confirmed to mediate 5-HT-induced contractions, and DA-9701 inhibited these 5-HT-induced contractions.

Smooth muscle cells pretreated with DA-9701 and then treated with 5-HT showed decreased immunoreactive protein bands compared to 5-HT-treated cells alone. These bands corresponded to 20-kDa phosphospecific Ser_19_-MLC_20_. These results suggest that DA-9701 inhibits phosphorylation of MLC_20._

In summary, 5-HT binds to its G protein-coupled receptors to induce smooth muscle contraction in feline ESMCs. 5-HT_2_, 5-HT_3_, and 5-HT_4_ receptors located on esophageal smooth muscle are coupled to G_αq_, G_αo_, and G_αs_. These G proteins activate PLC, which leads to Ca^2+^/calmodulin-dependent MLCK activation, resulting in MLC_20_ phosphorylation and cell contraction. DA-9701 inhibits 5-HT-induced contraction by inhibiting phosphorylation of MLC_20_.
